# Tag SNP selection for prediction of tick resistance in Brazilian Braford and Hereford cattle breeds using Bayesian methods

**DOI:** 10.1186/s12711-017-0325-2

**Published:** 2017-06-15

**Authors:** Bruna P. Sollero, Vinícius S. Junqueira, Cláudia C. G. Gomes, Alexandre R. Caetano, Fernando F. Cardoso

**Affiliations:** 1 Embrapa Pecuária Sul, Caixa Postal 242 - BR 153 - Km 633, Bagé, Rio Grande do Sul 96.401-970 Brazil; 2 Departamento de Zootecnia, Universidade Federal de Viçosa, Avenida Peter Henry Rolfs, s/n - Campus Universitário, Viçosa, Minas Gerais 36.570-000 Brazil; 3 Embrapa Recursos Genéticos e Biotecnologia, Parque Estacao Biologica Final Av. W/5 Norte, Brasilia-DF, C.P. 02372, Brasília, Distrito Federal 70770-917 Brazil; 4 Universidade Federal de Pelotas, Capão do Leão, Rio Grande do Sul 96.000-010 Brazil

## Abstract

**Background:**

Cattle resistance to ticks is known to be under genetic control with a complex biological mechanism within and among breeds. Our aim was to identify genomic segments and tag single nucleotide polymorphisms (SNPs) associated with tick-resistance in Hereford and Braford cattle. The predictive performance of a very low-density tag SNP panel was estimated and compared with results obtained with a 50 K SNP dataset.

**Results:**

BayesB (π = 0.99) was initially applied in a genome-wide association study (GWAS) for this complex trait by using deregressed estimated breeding values for tick counts and 41,045 SNP genotypes from 3455 animals raised in southern Brazil. To estimate the combined effect of a genomic region that is potentially associated with quantitative trait loci (QTL), 2519 non-overlapping 1-Mb windows that varied in SNP number were defined, with the top 48 windows including 914 SNPs and explaining more than 20% of the estimated genetic variance for tick resistance. Subsequently, the most informative SNPs were selected based on Bayesian parameters (model frequency and t-like statistics), linkage disequilibrium and minor allele frequency to propose a very low-density 58-SNP panel. Some of these tag SNPs mapped close to or within genes and pseudogenes that are functionally related to tick resistance. Prediction ability of this SNP panel was investigated by cross-validation using K-means and random clustering and a BayesA model to predict direct genomic values. Accuracies from these cross-validations were 0.27 ± 0.09 and 0.30 ± 0.09 for the K-means and random clustering groups, respectively, compared to respective values of 0.37 ± 0.08 and 0.43 ± 0.08 when using all 41,045 SNPs and BayesB with π = 0.99, or of 0.28 ± 0.07 and 0.40 ± 0.08 with π = 0.999.

**Conclusions:**

Bayesian GWAS model parameters can be used to select tag SNPs for a very low-density panel, which will include SNPs that are potentially linked to functional genes. It can be useful for cost-effective genomic selection tools, when one or a few key complex traits are of interest.

**Electronic supplementary material:**

The online version of this article (doi:10.1186/s12711-017-0325-2) contains supplementary material, which is available to authorized users.

## Background

Bovine ticks are endemic throughout some of the most productive livestock farming regions in the world [1]. In Brazil, the *Rhipicephalus* (*Boophilus*) *microplus* tick is one of the main causes of economic losses in cattle production and affects negatively the performance of their hosts both directly by blood sucking and indirectly as a vector of viral, bacterial and protozoal diseases [2]. Resistance to ticks is known to be under genetic control and the utility of genetic evaluations to classify cattle as resistant or susceptible based on natural tick infestations has already been demonstrated [3]. In addition, it is now well established that several biological mechanisms control host genetic resistance within and among breeds [4, 5]. Therefore, understanding the precise biological mechanisms that underlie vector–host–pathogen interactions is essential to develop innovative and sustainable tick management strategies [6].

The use of genome-wide single nucleotide polymorphism (SNP) panels of varying densities to detect statistical associations between phenotypes of interest and SNPs is a powerful method to identify the major genes that are involved in the control of complex traits. However, confounding factors, such as multicollinearity and estimability, which are embedded within multidimensional genotypic and/or phenotypic complex datasets must be considered, since it is necessary to weight the rate of false associations for the interpretation of results [7].

To date, several genomic regions associated with tick burden in dairy and/or beef cattle have been identified through association studies based on different regression methods [2, 8–14]. However, to estimate a greater proportion of the genetic variance explained by SNPs and to identify more complex relationships between SNPs, a shift to models that fit multiple SNPs simultaneously was proposed [15].

Bayesian methods provide a flexible approach to solve high-dimensional problems and enable simultaneous estimation of the effects of high-density SNPs [16]. The application of Bayesian inference methods in genome-wide association studies (GWAS) may improve the mapping of regions across the genome that contain causal variants, especially in the case of complex traits for which the majority of the SNPs each explain a small proportion of the total observed variance. Identification of the most informative SNPs associated with complex traits may contribute to the design of a low-density SNP panel with high predictive performance. This would be highly desirable since cost-effective solutions are needed for genomic selection to be implemented in most animal production sectors [17].

In this study, Bayesian methods were used on 50 K SNP panel data from Hereford and Braford cattle to identify genomic regions and tag SNPs associated with tick resistance. The predictive performance of the very low-density panel based on a selected subset of significant SNPs was estimated and compared with results obtained with the full SNP panel.

## Methods

### Animal sampling and data analyzed

All Hereford (HH) and Braford (BO) samples were derived from eight herds associated with the Delta G Connection breeding program (Rio Grande do Sul, Brazil). A subset of 3455 phenotyped animals was genotyped with the Illumina BovineSNP50 BeadChip. Total tick counts from one side of the body were recorded for each animal born between 2008 and 2011, two to three times consecutively, during the post-weaning period. In total, 10,673 tick counts were available for analyses. Variance components and breeding values were generated from log-transformed tick counts [3, 18] with the BLUPf90 family of programs [19] and estimated breeding values were used for GWAS analyses.

### Quality control analysis

SNP data quality control (QC) was performed using the R version 3.0.2/snpStats package [20] and the following criteria (thresholds): individuals for which the call rate was lower than 90%, heterozygosity deviations were above or below three standard deviations from the mean of the genotyped animals, with sex misidentifications and those that showed near-perfect collinearity with other individuals (>99.5%) were removed. Expected heterozygosity deviations were checked to identify individuals with either an excessive or reduced proportion of heterozygous genotypes, which may be indicative of DNA sample contamination or inbreeding, respectively. Individual SNPs were excluded from further analysis if their call rate was lower than 98%, their minor allele frequency (MAF) was lower than 3%, if they deviated significantly from Hardy–Weinberg equilibrium (Chi square test, P < 10^−7^) and if identical genotypes were found with other SNPs in neighboring positions. Moreover, only the SNP with the highest MAF was retained within groups of SNPs at the same position or that were highly correlated (>98%). A total of 41,045 SNPs (78%) and 3455 animals (98%) were retained for further analyses; these 3455 animals included 2803 BO and 652 HH and comprised yearling bulls, steers and heifers with respective phenotypes for tick count. Sporadically missing genotypes were imputed using FImpute software [21].

### Bayesian GWAS

Estimated breeding values (EBV) were obtained by adjusting a pedigree-based repeatability animal model to the tick count data. This model considered fixed effects for contemporary groups, regression coefficients with the linear additive effect for the zebu breed proportion, zebu–HH dominance effect, zebu–HH additive by additive epistatic effect [22], and linear and quadratic coefficients for animal age. Breed composition coefficients were derived from pedigree data [18]. Subsequently, deregressed estimated breeding values (DEBV) for tick resistance were calculated according to Garrick et al. [23], in order to remove parent average values and account for heterogeneous variance. It should be mentioned that the Hereford and Braford population studied here is evaluated and selected as a single breed-type with common breeding objectives and variance components by the Delta G Connection Breeding Program [24]. Moreover, as demonstrated by Biegelmeyer et al. [25], correlation of marker phase between these two breeds was estimated at 0.92 for SNPs less than 50 kb apart, which further supports the assumption that the initial detection analyses based on the 50 K SNP panel was suitable [18]. Therefore, we carried out a joint analysis that accounted for breed differences and heterosis to calculate DEBV. These pseudophenotypes, which did not include breed effects, were then analyzed with a model that includes random SNP allele substitution effects using the GenSel software version 4.0 [26]. Different Bayesian methods were applied to analyze DEBV data using genotypes as explanatory variables: BayesA, BayesB [16] and BayesCπ [27]. In BayesA and BayesB, each SNP is considered to have a locus-specific variance, which is derived from a scale inverted Chi square distribution *X* ^−2^ (*v*, *S*) with *v* = 4 degrees of freedom and a scale *S* = 0.0091. In addition, a prior distribution for the residual variance was also considered as *X* ^−2^ (*v*, *S*), but with *v* = 10 and scale *S* = 0.0572. Prior expected values of these Chi square distributions for the dispersion parameters that were equal to 0.0182 and 0.0715, respectively for the genetic and residual variances, were based on estimates previously obtained for tick counts in these BO and HH populations [18].

Prior specification for SNP effects in BayesB allows a proportion of the SNPs to have a zero effect, with a fixed probability π, while the remaining SNPs have normally distributed effects with a locus-specific variance and a probability 1-π. Conversely, in BayesA all SNP covariates are fitted, i.e., π = 0, for each Markov chain Monte Carlo (MCMC) cycle. The statistical model used for Bayesian analyses was: ${\mathbf{y}} = \sum\nolimits_{i = 1}^{k = 41,045} {\delta_{i} {\mathbf{z}}_{i} a_{i} + {\mathbf{e}}}$, where **y** is a vector of phenotypes (DEBV); *k* is the total number of SNPs; *δ*_*i*_ indicates whether SNP *i* is included in (*δ*_*i*_ = 1) or excluded (*δ*_*i*_ = 0) from the model for a given iteration of the MCMC; **z**_*i*_ is a vector of genotypes of the fitted SNP *i*, coded −10/0/10; *a*_*i*_ is the random substitution effect of the fitted SNP *i* with its own variance $\sigma_{{a_{i} }}^{2}$ and an a priori zero effect with probability π or a non-zero effect with probability 1-π, and **e** is the vector of normally distributed random residuals. In BayesCπ, the probability that a SNP has a zero effect was treated as unknown and a common effect variance was assumed for all the SNPs having a non-zero effect, while for BayesA *δ*_*i*_ was always equal to 1. Initial SNP effects were estimated for all individuals with BayesCπ (setting π to 0.5 a priori and as starting value) as proposed by Sun et al. [28] and de Oliveira et al. [29]. Subsequent analyses with BayesB tested the posterior mean of π obtained with BayesCπ and π = 0.99. A total of 41,000 chain iterations was used, of which the first 1000 were discarded as burn-in. Convergence of MCMC chains was verified by the Geweke test [30] using the boa (Bayesian output analysis) R package [31].

### Top windows and tag SNPs

SNPs were allocated to 2519 non-overlapping 1-Mb genome windows that contained different numbers of SNPs based on the physical map order derived from the bovine genome assembly UMD3.1 [32]. Genetic variance explained jointly by each SNP subset, considered as window variance, was estimated and subsequently converted into the proportion of total genetic variance explained by the window [28, 33].

Genome regions that potentially contained quantitative trait loci (QTL) associated with tick resistance, referred to as top windows, were identified based on a threshold that is defined in terms of genetic variance contribution as described by Schurink et al. [34]. Top windows were identified in the GWAS by considering all 3455 animals and 41,045 SNPs and by applying the BayesB method (π = 0.99). Assuming an equal contribution of all genomic regions, the expected proportion of genetic variance explained by each of the 2519 windows was equal to 0.04%. Hence, 1-Mb size windows that explained at least 0.2% of the genetic variance, which corresponds to five times the expected variance (0.04% × 5 = 0.2%), were considered as putative QTL [35, 36] and selected for further analyses.

To identify potential SNPs to construct a low-density panel, a tag SNP selection strategy was tested within the top windows by considering model frequency (MF), *t*-like statistic (TL), linkage disequilibrium (LD) and minor allele frequency (MAF) parameters. In GenSel, MF reflects the proportion of post-burn-in iterations that included that particular covariate (SNP) in the model, while TL is the absolute value of posterior mean effects (for only those chains that included the SNP in the model) divided by the respective standard deviations of those effects. The R/snpStats package [20] was used to obtain LD values and the R/LDheatmap package [37] was applied to generate plots of LD in relation to physical distances.

We selected SNPs with the maximum MF within each top window as top SNPs. Then, we also selected all SNPs within top windows that had MF values above the minimum observed MF value for top SNPs. This step aimed at selecting SNPs that were not at the top of their own windows, but that had sufficiently large MF to exceed the MF value of the top SNPs located in other selected windows. A similar approach was used to evaluate consistency of SNP effects by considering TL. Within those pre-selected SNPs based on MF, the minimum TL value was determined and set as the threshold to select the remaining SNPs within top windows that exceeded this minimum TL value. The final step to construct the tag SNP panel aimed at removing redundant SNPs due to observed high LD among subsets of SNPs pre-selected by MF and TL. Thus, when two SNPs were observed with r^2^ values higher than 0.4 [38], only the SNP with the highest MAF was retained.

### Prediction ability of selected tag SNP panels

To check the effectiveness of choosing only the most informative SNPs for genetic prediction of tick resistance, genotypic and tick count data from 3455 animals were divided into five sub-groups based on two strategies: K-means clustering according to SNP relationship distance, or randomly, using the R 3.0.2/base package. Cross-validation was carried out within the grouping strategy by selecting subsets of SNPs as described above using data from four of the five groups and then testing the derived tag SNP panel for genomic prediction in the group that was not included in the selection process.

For individuals within each testing group, direct genomic values (DGV) were calculated based on their tag SNP genotypes and corresponding allele substitution effects estimated from training data, which consisted in data on tick counts and genotypes from the four other groups. In this step, we used the BayesA method, such that all selected SNPs had non-zero effects. For the *j*th individual:

$$\widehat{DGV}_{j} = \mathop \sum \limits_{i = 1}^{K} z_{ji} \hat{a}_{i} ,$$

where the estimated SNP effect, $\hat{a}_{i}$, is represented by its posterior mean obtained by the BayesA method, and *z*_*ji*_ represents the genotype for the *i*th SNP from the total *K* SNPs included in the very low-density panel.

Pooled prediction accuracies of DGV were derived from their genetic correlations with tick count data in a bivariate analysis using a within-group pedigree-based numerator relationship matrix (${\mathbf{A}}^{*}$; [39]) and were computed using the Gibbs2f90 software [19]. For our fivefold cross-validation:

$${\mathbf{A}}^{*} \left[ {\begin{array}{*{20}c} {{\mathbf{A}}_{11} } & \cdots & 0 \\ \vdots & \ddots & \vdots \\ 0 & \cdots & {{\mathbf{A}}_{55} } \\ \end{array} } \right],$$

**A**_*cc*_ is the numerator relationship matrix within cluster *c*.

Prediction accuracies were also estimated within each cluster *c*, as proposed by Legarra et al. [40]. Additional details of this cross-validation approach have been described by Cardoso et al. [18] for the full set of 41,045 SNPs.

To further check the effectiveness of our selection process, prediction accuracies of DGV were also obtained with the same fivefold cross-validation with BayesB considering all 41,045 SNPs and π = 0.999. With this model, the built-in selection process fits, within each cycle, a number of SNPs that is comparable to that included in our proposed panel (π ≈ 1 − *n*_tagSNP_/41,045).

### Functional analysis

To map tag SNPs to genes and genomic regions, the BEDTools software [41] was used to relate SNP data with the *Bos taurus* genome information provided by the Ensembl database [42]. Alternatively, for the SNPs that were not mapped within any known gene within ±100 kb on the *Bos taurus* genome, the package NCBI2R [43] was used to search for the closest known genes in the genome of other species. Using DAVID bioinformatics resources [44], the biological meaning of the genes mapped to tag SNPs was extracted. The online software STRING v9.1 [45] was used to identify potential protein–protein interactions related to the identified genes.

## Results and discussion

### Groups of animals

Genomic relationships between the five groups of animals based on K-means clustering and the number of individuals in each group are in Table 1. Each of the five groups that were obtained from random distribution contained 691 animals and displayed similar relatedness within and across groups.

**Table 1:** Number of individuals (N) and average (±SD) zebu proportions, and within- and between-group genomic relationships (Gij) for the K-means clustering groups

Group	N	Zebu proportion	Within-group Gij	Between-group Gij
1	629	0.02	0.140 ± 0.04	−0.030 ± 0.04
2	230	0.37	0.070 ± 0.05	0.005 ± 0.05
3	1211	0.35	0.004 ± 0.03	0.003 ± 0.03
4	471	0.34	0.010 ± 0.04	0.002 ± 0.03
5	914	0.35	0.020 ± 0.03	0.010 ± 0.04

The majority of the Hereford breed animals were clustered into Group 1

### Choice of π

The BayesCπ analysis that included all animals and SNPs simultaneously resulted in a posterior (π) of 0.9999 and therefore, only approximately four SNPs (0.01%) were fitted in each iteration of the MCMC chain. Using π = 0.9999 in a BayesB analysis resulted in a very low estimated heritability (*h* ^2^ = 0.02), which corresponded to a small fraction of the pedigree-based heritability (*h* ^2^ = 0.19) obtained with the same dataset [18], and was similar to the lower-bound heritability estimates recently reported for cattle tick resistance [14] in a GWAS that analyzed *A. hebraeum* tick counts on the tail of South African Nguni cattle (0.02). Some cycles contained no fitted SNPs when an extremely high value of π (0.9999) was used in BayesB, which resulted in the absence of any predictive SNPs, and thus this model contributed mostly to the estimated residual variance. These results suggest that BayesCπ could not estimate π appropriately based on the present data.

Subsequently, more SNPs were fitted in the BayesB model by setting π = 0.99 for the GWAS including all animals simultaneously and for each group in the cross-validation process. With this new π value and the full GWAS data, the proportion of variance explained by SNPs increased to 0.1132, which corresponds to 58% of the estimated heritability based on pedigree-based analysis of this dataset on tick resistance [18]. This reduced genomic heritability may result from incomplete linkage disequilibrium between the SNPs studied and the QTL affecting the trait [46], when only 1% of the markers were fit in each chain cycle (π = 0.99). Alternatively, the proportion of phenotypic variance explained by SNPs when fitting BayesA with the full SNP panel (0.1755) was much closer to that based on pedigree analysis (0.19). These BayesA and pedigree estimated heritabilities were higher than that reported by Porto Neto et al. [13] for the analysis of tick burden in Brahman cattle (0.09). Setting π at 0.99, in spite of the lower estimated heritability compared to BayesA or pedigree analysis, has the advantage of fitting only the regions in strong association with the trait [33, 35, 47]. According to Fernando and Garrick [48], higher values of π can be more discriminating for the identification of the largest QTL, which is an important factor for selecting tag SNPs. Moreover, it was shown that the SNP-specific variances in BayesB led to less shrinkage for SNPs with the largest effects compared to BayesC [27].

All Bayesian GWAS analyses were visually checked and passed the Geweke’s test for convergence.

### Top windows and QTL detection

**Fig. 1:**
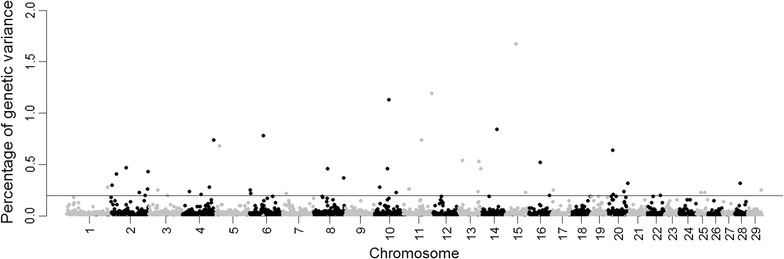
Manhattan plot displaying Bayesian genome-wide association estimates (BayesB, π = 0.99) for tick resistance. The *Y-axis* represents the proportion of the total genetic variance explained by 1-Mb windows across the bovine genome and the *X-axis* represents the chromosomal location of windows (2519 non-overlapping windows). Windows explaining more than 0.2% of the genetic variance are above the *grey line*

The proportion of genetic variance explained by each of the 2519 1-Mb windows including all 41,045 SNPs across the genome is shown in Fig. 1. The number of SNPs included in the windows varied from 1 (only 10 windows) to 30. Forty-eight windows represented by 914 SNPs were found to jointly explain more than 20% of the genetic variance and were considered as top windows containing QTL (Table 2).

**Table 2:** Windows explaining the largest percentages of tick resistance genetic variance in Hereford and Braford cattle breeds

Obs	Window	Start SNP name	End SNP name	N SNP	%Var	Cum Var	chr_Mb	Top SNP name	ModelFreq	t.like	Stand_effect
1	1621	ARS-BFGL-BAC-27751	ARS-BFGL-NGS-115263	17	1.67	1.67	15_37	ARS-BFGL-NGS-5811	0.7574	1.393	1.4036
2	1319	ARS-BFGL-NGS-112243	ARS-BFGL-NGS-12954	17	1.19	2.86	11_101	ARS-BFGL-NGS-111179	0.6731	1.243	1.1350
3	1164	ARS-BFGL-NGS-1854	ARS-BFGL-NGS-24556	27	1.13	3.99	10_51	Hapmap58695-rs29019899	0.5048	1.076	0.8828
4	1553	ARS-BFGL-NGS-115527	ARS-BFGL-NGS-1112	15	0.84	4.83	14_54	BTB-00915241	0.3045	0.982	0.4614
5	710	BTB-01688071	ARS-BFGL-NGS-58275	23	0.78	5.6	6_49	BTB-02002785	0.3176	0.986	0.4781
6	1283	ARS-BFGL-NGS-44192	Hapmap60779-rs29022104	14	0.74	6.35	11_65	Hapmap60779-rs29022104	0.3697	1.005	0.6143
7	531	Hapmap57291-ss46526771	Hapmap22875-BTA-155031	14	0.74	7.09	4_113	Hapmap22875-BTA-155031	0.4834	1.06	0.8291
8	553	Hapmap36482-SCAFFOLD163485_1458	BTA-87049-no-rs	12	0.68	7.77	5_14	Hapmap52967-rs29017027	0.4047	1.022	0.6210
9	1974	ARS-BFGL-NGS-13160	BTB-00774670	20	0.64	8.4	20_17	Hapmap34041-BES1_Contig298_838	0.327	0.988	0.5321
10	1429	Hapmap48542-BTA-97857	ARS-BFGL-NGS-27497	11	0.54	8.95	13_14	Hapmap44228-BTA-34185	0.3342	0.99	0.4960
11	1488	ARS-BFGL-NGS-107401	ARS-BFGL-NGS-4602	18	0.53	9.48	13_73	Hapmap40517-BTA-33731	0.3514	0.997	0.5201
12	1709	Hapmap54735-ss46526095	Hapmap56619-rs29009970	16	0.52	10	16_40	ARS-BFGL-NGS-40365	0.2408	0.956	0.3808
13	214	Hapmap25908-BTA-160304	Hapmap60963-rs29015781	26	0.47	10.47	2_55	ARS-BFGL-NGS-111213	0.1665	0.937	0.2428
14	1159	ARS-BFGL-NGS-113665	ARS-BFGL-NGS-10383	21	0.46	10.93	10_46	ARS-BFGL-NGS-60054	0.2224	0.953	0.3154
15	944	Hapmap41647-BTA-81135	ARS-BFGL-NGS-111988	20	0.46	11.39	8_50	BTB-01398754	0.0986	0.902	0.1449
16	1495	ARS-BFGL-NGS-83969	Hapmap41120-BTA-99310	20	0.46	11.85	13_80	Hapmap41120-BTA-99310	0.1838	0.942	0.2532
17	293	ARS-BFGL-NGS-22691	ARS-BFGL-NGS-17681	22	0.43	12.28	2_134	ARS-BFGL-NGS-113378	0.2608	0.956	0.4564
18	179	ARS-BFGL-NGS-39206	BTB-01168392	21	0.41	12.69	2_20	BTB-00082871	0.2273	0.953	0.3302
19	1001	Hapmap25843-BTA-146186	ARS-BFGL-NGS-20859	18	0.37	13.07	8_107	Hapmap40677-BTA-121871	0.1692	0.936	0.2455
20	2440	BTB-00980670	Hapmap34915-BES7_Contig278_1082	17	0.32	13.39	28_20	BTB-01129090	0.1208	0.923	0.1530
21	2028	Hapmap44700-BTA-34998	ARS-BFGL-NGS-118166	15	0.32	13.71	20_71	ARS-BFGL-NGS-13702	0.2361	0.947	0.3888
22	163	Hapmap43083-BTA-86781	BTA-47785-no-rs	17	0.3	14.01	2_4	BTB-00077766	0.1948	0.944	0.2567
23	1132	ARS-BFGL-NGS-94247	ARS-BFGL-NGS-32828	24	0.28	14.29	10_18	ARS-BFGL-NGS-107048	0.2059	0.947	0.3001
24	147	Hapmap30204-BTA-124882	BTB-01761180	28	0.28	14.57	1_147	BTB-01301015	0.1687	0.934	0.2423
25	515	Hapmap25270-BTA-142450	ARS-BFGL-NGS-12738	11	0.28	14.85	4_97	Hapmap25270-BTA-142450	0.1796	0.931	0.2960
26	1239	Hapmap43962-BTA-86597	Hapmap42711-BTA-87541	24	0.26	15.11	11_21	BTB-00464454	0.142	0.926	0.2100
27	1238	ARS-BFGL-NGS-20053	BTB-00464777	19	0.26	15.37	11_20	BTA-104373-no-rs^a^	0.1533	0.922	0.2280
28	290	ARS-BFGL-NGS-54356	ARS-BFGL-NGS-77887	26	0.26	15.63	2_131	BTB-00117780	0.1469	0.931	0.1938
29	664	Hapmap30828-BTA-143720	Hapmap30881-BTA-159706	23	0.25	15.88	6_3	Hapmap30881-BTA-159706	0.171	0.937	0.2465
30	2515	Hapmap24672-BTA-140771	ARS-BFGL-NGS-29493	27	0.25	16.13	29_48	BTA-66199-no-rs	0.211	0.951	0.2818
31	329	ARS-BFGL-NGS-117560	Hapmap51025-BTA-67309	19	0.25	16.38	3_33	ARS-BFGL-NGS-119309	0.0962	0.91	0.1189
32	1758	ARS-BFGL-NGS-5880	BTA-122662-no-rs	23	0.25	16.63	17_7	ARS-BFGL-BAC-27352	0.1857	0.944	0.2496
33	2013	ARS-BFGL-NGS-55465	Hapmap51244-BTA-50863	19	0.24	16.87	20_56	Hapmap43377-BTA-85612	0.1385	0.927	0.1717
34	442	ARS-BFGL-NGS-113848	BTB-00169886	16	0.24	17.12	4_24	Hapmap45129-BTA-72713	0.1427	0.925	0.2042
35	1485	ARS-BFGL-NGS-118627	ARS-BFGL-BAC-15769	17	0.24	17.35	13_70	Hapmap57013-rs29019369	0.1502	0.934	0.1073
36	1656	ARS-BFGL-BAC-18252	Hapmap45825-BTA-25376	16	0.23	17.58	15_72	Hapmap45825-BTA-25376	0.1828	0.943	0.2411
37	261	Hapmap48190-BTA-114376	BTA-48503-no-rs	11	0.23	17.81	2_102	Hapmap36094-SCAFFOLD96944_22403	0.1323	0.928	0.1683
38	1190	ARS-BFGL-NGS-38839	Hapmap50492-BTA-86239	16	0.23	18.04	10_77	ARS-BFGL-NGS-111871	0.1883	0.945	0.2477
39	2294	ARS-BFGL-NGS-26313	ARS-BFGL-NGS-34801	17	0.23	18.26	25_15	ARS-BFGL-NGS-84660	0.1403	0.933	0.1543
40	2309	ARS-BFGL-BAC-3777	ARS-BFGL-BAC-47171	21	0.23	18.49	25_30	ARS-BFGL-BAC-37178	0.1584	0.911	0.3051
41	665	ARS-BFGL-NGS-56212	BTB-01468045	20	0.22	18.71	6_4	BTB-01280976	0.0959	0.922	0.1089
42	794	ARS-BFGL-NGS-93802	Hapmap57279-ss46526160	19	0.22	18.93	7_13	ARS-BFGL-NGS-111257	0.0898	0.91	0.1073
43	484	ARS-BFGL-NGS-26541	ARS-BFGL-NGS-44674	20	0.21	19.14	4_66	ARS-BFGL-NGS-36591	0.1501	0.932	0.2019
44	1976	BTB-00775794	Hapmap49633-BTA-50009	16	0.21	19.35	20_19	Hapmap28040-BTA-134983	0.1529	0.935	0.2100
45	1743	BTB-00661933	ARS-BFGL-NGS-99802	17	0.2	19.56	16_74	Hapmap48746-BTA-40116	0.1664	0.938	0.2172
46	282	ARS-BFGL-NGS-102874	ARS-BFGL-NGS-15468	22	0.2	19.76	2_123	ARS-BFGL-NGS-102874	0.1163	0.915	0.1622
47	2146	ARS-BFGL-NGS-118471	Hapmap38075-BTA-54630	18	0.2	19.96	22_45	BTB-00849206	0.1658	0.922	0.2876
48	364	BTA-68264-no-rs	Hapmap44273-BTA-68311	24	0.2	20.15	3_68	ARS-BFGL-NGS-33433	0.1601	0.912	0.2923

*Obs* sequence number of the top 48 1-Mb non-overlapping windows, *Window* window coded number by GenSel according to physical map order, *Start SNP name* name of the first SNP flanking the window, *End SNP name* name of the last SNP flanking the window, *N SNP* Number of SNPs within the window, *%Var* percentage of genetic variance explained by the window, *Cum Var* cumulative percentage of genetic variance, *chr_Mb* BTA autosome and position of the window in Mb pairs, *Top SNP name* SNP name of the top SNP in the window (in terms of *ModelFreq* and or *t.like* statistics) and respective values of *ModelFreq*, *t.like* and standard effect (Stand_effect) of each top SNP

^a^This SNP was not included in the low-density panels proposed because it is in LD with BTB-00464454 and it has a low MAF

Some of the detected windows coincided with previously reported QTL from linkage analyses and GWAS for tick burden (Cattle QTL database, [49]), i.e. on BTA2 (BTA for *Bos taurus* chromosome) top windows number 163 located at 4 Mb (identified according to the first SNP position in the window) and number 214 at 55 Mb, top windows number 364 at 68 Mb on BTA3, number 553 at 14 Mb on BTA5, number 794 at 13 Mb on BTA7, number 1190 at 77 Mb on BTA10, number 1283 at 65 Mb on BTA11, and number 1553 at 54 Mb on BTA14 (Table 2).

The first 12 top windows jointly explained more than 10% of the genetic variance and three genomic regions (top three windows) individually explained more than 1% of the genetic variance for tick resistance (Table 2). In these three regions (BTA15 at 37 Mb, BTA11 at 101 Mb and BTA10 at 51 Mb), within ±100 kb on each side of the SNPs included in the respective top windows, four SNPs (rs110197574 and rs41665212, rs29019899 and rs110144789) were mapped to annotated genes or pseudogenes in the bovine or human genomes (see Additional file 1). Two SNPs on BTA15, were located at ~40 kb apart from each other (rs110197574 and rs41665212) and mapped to HSA5 (HSA for *Homo sapiens* chromosome) close to the *RPS15P8* pseudogene (*ribosomal protein S15*, *pseudogene 8*). Other positional candidate genes close to SNP rs110144789 (BTA11) are *LAMC3* (*laminin*, *gamma 3*), *ABL1* (*ABL proto*-*oncogene 1*, *non*-*receptor tyrosine kinase*), *FIBCD1* (*fibrinogen C domain containing 1*), *QRFP* (*pyroglutamylated RFamide peptide*); and to rs29019899 (BTA10) are *ADAM10* (*metallopeptidade domain 10*), *LIPC* (*lipase*, *hepatic*) and the gene *5S_rRNA* (*ENSBTAG00000037226*, *5S ribosomal RNA*); all these genes are annotated on the bovine genome sequence.

The SNP with the largest effect on tick count (rs110197574) was mapped to the *RPS15P8* gene, which in humans encodes a ribosomal protein that is a component of the 40S subunit [50]. Analysis of the genes that encode components of the ribosome or proteins involved in ribosome biosynthesis is very complex, and considering the wide range of biological processes in which ribosomal genes may be involved, the potential role of *RPS15P8* in tick resistance needs to be further investigated. Barendse [10] reported a polymorphism in the *RPS13* (*ribosomal protein S13*) gene that is associated with increased tick resistance in cattle. The *ADAM10* (*ADAM metallopeptidase domain 10*) gene (BTA10) encodes a characterized member of the *ADAM*-family of metalloproteases, which has a prominent role in inflammation [51]. Furthermore, different inflammatory responses can activate ADAM10-mediated proteolysis of E-cadherin, which is a prime mediator of epithelial cell-to-cell interactions, in primary human keratinocytes and in diseased human skin [52]. According to Porto Neto et al. [53] at approximately 15 Mb on BTA10, some locus-haplotypes that include SNPs in the *ITGA11* (*integrin alpha 11*) gene are associated with tick burden in dairy cattle breeds (Australian Red, Brown Swiss, Channel Isle, Holstein and composites) and Brahman beef cattle. Although this gene is functionally described as related with cellular adhesion control, these authors suggested that it had a role in modulating cellular immune responses. Both of these genes (*ADAM10* and *ITGA11*) are on BTA10 and may be involved in the control of cellular adhesion and migration during the process of skin infection caused by tick burden. Other studies based on microsatellite whole-genome scans [2, 54] and a GWAS with a low-density SNP panel [10] also reported QTL associated to tick burden on BTA10. In agreement with Regitano et al. [54], we identified potential QTL at 18 Mb on BTA10, as well as on BTA4 (97 Mb). On BTA10, beyond the region that contains the *ADAM10* gene (~50 Mb), three other top windows (Table 2) were detected as potential QTL in agreement with Machado et al. [2].

Anaplasmosis is an infectious rickettsial disease (*Anaplasma marginale*) that is mainly transmitted by ticks [55] and negatively impacts cattle production in tropical and subtropical areas [56]. In humans, *A. phagocytophilum*, an obligatory intracellular parasite of human granulocytes, causes a similar disease and was shown to activate the ABL1 signaling pathway during cell invasion. This protein is critical for intracellular invasion and infection establishment. Thus, a novel strategy for the treatment of human granulocytic anaplasmosis was proposed through inhibition of the host cell Abl-1 signaling pathway [57]. In addition to being possibly directly associated with tick resistance, results that we obtained from the analysis with STRING suggest the occurrence of interactions between the *ABL1* gene and other genes that are associated with the most informative SNPs found to affect tick count (*LAMC3* or *PLCG1*, *phospholipase C*, *gamma 1* on BTA13; *CDC42*, *cell division cycle 42* on BTA2; *SDC3*, *syndecan 3* on BTA2 and *EPS8L3*, *epidermal growth factor receptor kinase substrate 8*-*like protein 3* on BTA3), which indicates that a gene network may be involved in cattle resistance to ticks. Two other top windows on BTA11 with putative QTL were also reported by Machado et al. [2].

Other genomic regions on BTA17 at 7 Mb flanked by SNPs ARS-BFGL-NGS-5880 and BTA-122662-no-rs (top window 1758, Table 2) also include two SNPs (rs43499108 and rs29011077), which have been reported to be associated with *R. evertsi evertsi* tick count in African cattle [14]. The top SNP in this window (rs109822497) was mapped to the *double cortin*-*like kinase 2* (*DCLK2*) gene, near the *LRBA* gene, which is suggested by these authors to be associated with protein kinase A that supports the secretion and/or membrane deposition of immune effector molecules.

### Selecting tag SNPs

Based on the model frequency (MF) and *t*-like statistic (TL) provided by GenSel, in the strategy used for tag SNP selection, a minimum MF value of 0.0898 was determined among all top SNPs representing each of the 48 top windows. Nine additional SNPs with an MF above this threshold were selected from the list of 914 SNPs within the top windows. Within those 57 (48 + 9) pre-selected SNPs, the minimum observed TL of 0.902 was set as another threshold to select SNPs within the 914-SNP list that exceeded this lower bound TL value. The subset of SNPs that were pre-selected based on MF and TL contained 63 SNPs, which were subsequently analyzed in terms of LD and MAF, resulting in a final list of 58 SNPs. These selected SNPs were distributed on most of the bovine chromosomes, except BTA9, 12, 18, 19, 21, 23, 24, 26 and 27. It is interesting to mention that nine of the 58 SNPs were located on BTA2. Previous studies identified significant allele effects associated with tick burden in a GWAS analysis, as well as positional candidate genes on chromosome BTA2 [2, 10, 11, 58]. Our proposed panel included SNPs that represented 47 of the 48 top windows, because SNP rs43669951 was included in two adjacent windows on BTA11. The resulting minimum MF value among the SNPs in this panel was equal to 0.0744 and only two windows included three SNPs on BTA28 at ~20 Mb and BTA13 at ~80 Mb, while all other windows included only one or two SNPs.

This strategy to select more informative SNPs that are uniquely linked to QTL related to cattle tick resistance within top windows favored those that were more often included in the Bayesian mixture model (greater MF) and with a more consistent effect (greater TL), but avoided redundancy due to LD. Based on that, our goal was to retain SNPs that had a suitable prediction ability to build very low-density panels for cost-effective genomic selection of tick resistance in cattle.

The proportion of fitted models that included a SNP and used it to infer associations with the phenotype under study, represented by MF [28, 59], was highly correlated (*r* = 0.99) to the SNP adjusted effect, $\left( {\hat{a}_{i} } \right)^{2} /\text{var} \left( {\hat{a}_{i} } \right)$, in the subset of 914 SNPs. In contrast, a moderate correlation was found between MF and TL (*r* = 0.46). Since TL is an alternative measure of SNP effect (i.e. $\left| {\hat{a}_{i} } \right|/{\text{sd}}\left( {\hat{a}_{i} } \right)$ is calculated by considering only the cycles in which SNP *i* was included in the model) and due to its incomplete correlation with MF, we were able to combine both parameters, MF and TL, to select informative SNPs for which the estimated effects were consistent [26].

According to some authors [33, 36], SNPs with an MF higher than 0.90 are deemed significant in a Bayesian GWAS analysis, and those with an MF lower than 0.10 represent false positives. In the current study, the highest MF for a top SNP was 0.7574 for rs110197574/ARS-BFGL-NGS-5811 located on BTA15 within the 1-Mb window that explained the greatest proportion of the genetic variance (Table 2). Therefore, this particular SNP had a non-zero effect in 75% of MCMC samples. Considering all SNPs with the highest MF within each of the 48 top windows according to our BayesB analysis (Table 2), the average MF was equal to 0.23 ± 0.14. This result indicates that there are no major genes affecting tick resistance and that most of the SNPs each explained a small proportion of the phenotypic variation for this trait. Similar results were reported by other authors who concluded that selection programs must use SNP panels rather than single SNPs with high predictive value [60]. This emphasizes the fact that it cannot be expected to find a very small number of genes with a large effect, which would lead to accurate prediction for tick resistance. In this regard, our approach was to identify a minimal set of informative SNPs that would still yield useful predictions compared to those derived from high-density SNP panels, but potentially reducing genotyping costs.

**Fig. 2:**
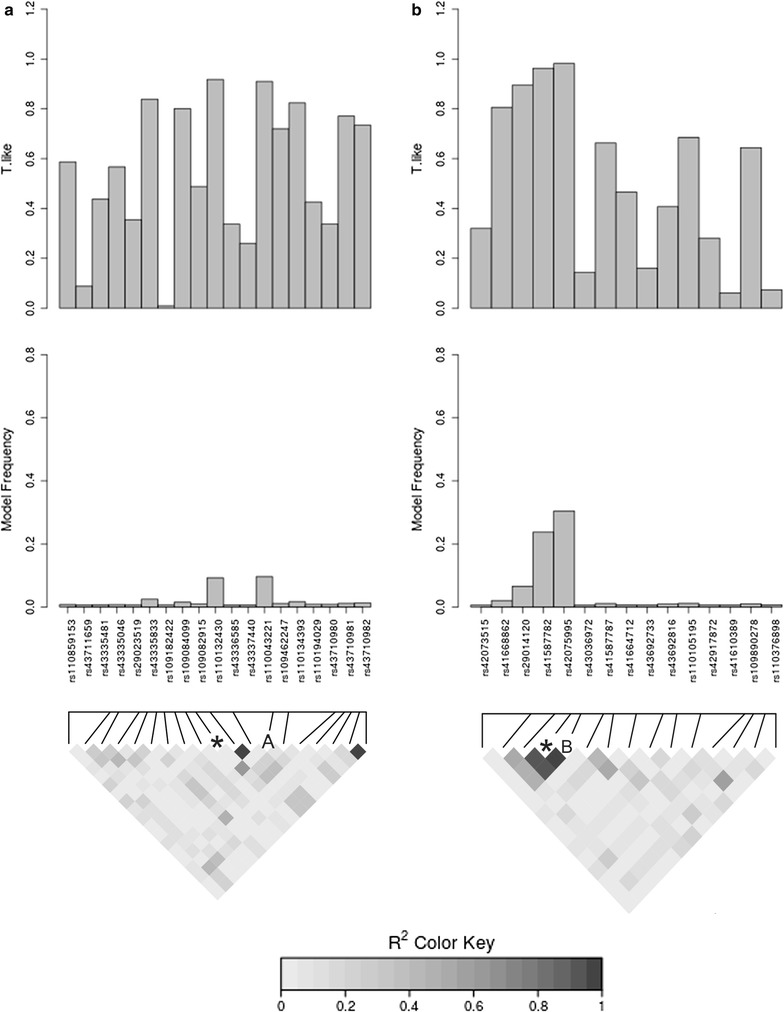
MF and TL estimates and LD heatmaps, for neighboring SNPs in two windows (1 Mb) according to physical map order. **a** Top window on BTA3. *Markers excluded by LD parameter. “A” Marker selected as tag SNP in the low-density panel. **b** Top window on BTA14. *Markers excluded by LD parameter. “B” Marker selected as tag SNP in the low-density panel

Figure 2 shows LD-heatmaps and respective MF and TL values for two distinct windows, which highlight SNPs in the proposed list of 58 SNPs. The first window, on BTA3 at ~33 Mb (Fig. 2a), represents a chromosomal region that contains the SNP ARS-BFGL-NGS-119309 (rs110043221) selected as its tag SNP. Figure 2a also illustrates the case of the ARS-BFGL-NGS-77834 (rs110132430) SNP that has TL and MF values higher than the selection threshold, but that was excluded because it was in LD (r^2^ > 0.4) with another SNP with a higher MF (rs110043221). SNPs within this window were mapped to a bovine genomic region that contains three genes, *EPS8L3* (*EPS8*-*like 3*), *GSTM1*/*3* (*glutathione S*-*transferase mu 1 and 3*), *CSF1* (*colony stimulating factor 1*—*macrophage*) and a microRNA bta-mir-2413. De Rose et al. [61] showed that cytokines, such as the granulocyte and macrophage colony stimulating factor (GM-CSF) or interleukin (IL)-1b have increased vaccine effectiveness by enhancing the immune response against *Rhipicephalus* (*Boophilus*) *microplus* in sheep. The second top window with an effect on tick resistance is located at ~54 Mb on BTA14 (Fig. 2b), contains 15 SNPs and includes an LD block represented by SNP BTB-00915241 (rs42075995). In this case, SNP BTA-60194-no-rs (rs41587782) was in high LD with the representative tag SNP and thus, was excluded in the final step of the selection strategy. The differential pattern of MF and TL variation of SNPs was critical to effective tag SNP selection, since the top SNPs were clearly distinct in the histograms of those windows (Fig. 2). Therefore, most of the SNPs with low MF/TL were excluded in the first two selection steps and the remaining ones were evaluated in terms of LD/MAF in a final step with only a few additional exclusions.

The genes that map to the regions containing the 58 SNPs that were selected to compose the proposed very low-density panel are listed in Additional file 1. One hundred and three genes are located in the genome regions that are on either side of 52 of these SNPs, based on the information derived from the bovine (43 SNPs) and human (9 SNPs) genomes. Gene ontologies and biological pathways which may be related to the biological processes that underlie vector-host-pathogen interactions, such as pathways involved in inflammation mediated by chemokine and cytokine signaling, cell receptor signaling and calcium signaling, were identified for these genes. Also, enrichment analysis identified genes that are associated with biological processes such as regulation of adaptive immune response (e.g. *ADA*), activation of immune response (e.g. *ABL*-*1*), positive regulation of macrophage derived from cell differentiation (e.g. *CSF1*), regulation of inflammatory response and leukocyte chemotaxis (e.g. *ADAM10*), cell–cell junction organization (e.g. *CDC42*) and leukocyte activation involved in immune response (e.g. *ADA*, *ABL*-*1*).

### Prediction ability of tag SNP panels

The proposed BayesB (π = 0.99) GWAS and tag SNP selection strategy was applied to each of five K-means and five random cross-validation subsets and generated 10 alternative SNP panels, which included 47 to 86 SNPs (Table 3). Three hundred and fifty unique SNPs were selected based on the combination of all 10 tag SNP panels derived by the cross-validation analyses. The number of times that each of the original 58 tag SNPs (our proposed panel using the whole data) was represented in those 10 cross-validation subsets is presented in Additional file 1.

**Table 3:** Posterior mean proportion of variance explained by markers (*h* ^2^) using different Bayesian methods, and number of chromosome segments and SNPs involved in the very low-density panel selection by K-means and random cross-validation group

Group	*h* ^2^				SNP panel selection		
	BayesB π = 0.99	BayesB π = 0.999	BayesA full	BayesA tag	Top windows^a^	Top SNPs^b^	Tag SNPs^c^
K-means 1	0.13	0.06	0.19	0.10	41	741	47
K-means 2	0.10	0.04	0.17	0.09	46	878	57
K-means 3	0.12	0.05	0.18	0.12	39	727	67
K-means 4	0.12	0.05	0.18	0.11	48	941	79
K-means 5	0.11	0.04	0.18	0.10	43	799	55
Random 1	0.12	0.05	0.18	0.11	42	778	57
Random 2	0.11	0.04	0.18	0.12	53	956	70
Random 3	0.11	0.05	0.18	0.13	52	1008	86
Random 4	0.12	0.05	0.18	0.11	48	900	78
Random 5	0.11	0.04	0.18	0.12	55	1005	79

^a^Top windows represents the number of windows that explained above 0.2% of the genetic variance in the BayesB (π = 0.99) GWAS analysis

^b^Top SNPs represents the number of SNPs included in those top windows

^c^Tag SNPs represents the number of SNPs selected as more informative according to the criteria based on model frequency and t.like statistics, linkage disequilibrium and minor allele frequency

The posterior proportions of the phenotypic variance, which was explained by the SNPs, i.e. the SNP-heritabilities (*h* ^2^), that were estimated with BayesA using the very low-density panel derived for each of the 10 cross-validation groups ranged from 0.09 to 0.12, and were very similar to the *h* ^2^ estimated with BayesB (π = 0.99) using the full set of 41,045 SNPs (Table 3). Conversely, the *h* ^2^ obtained with BayesB also using all 41,045 SNPs but with π = 0.999 (~1–58/41,045) were lower and ranged from 0.04 to 0.06 (Table 3). These results demonstrate that a very small number of SNPs selected based on the tag-method explains more variation than a similar number of SNPs chosen with the Bayes-B method (on average 0.14% of the total number of available 41,045 SNPs).

Bayesian approaches generally combine shrinkage procedures to consider different variances for individual SNPs and mixture models, in which the prior information about the distribution of SNP effects is used to coerce negligible effects towards zero. In the case of tag SNP panels, BayesA was chosen because these panels are expected to include only the most significant SNPs each with a detectable effect (π = 0), while allowing for SNPs to have specific variances and consequently different effect sizes [16].

The effectiveness of the applied strategy for selecting more informative SNPs for genomic prediction of cattle tick resistance was assessed by pooled breeding value prediction accuracies measured as the genetic correlation between cross-validation DGV and tick count data, which were equal to 0.27 ± 0.09 for the K-means clustering groups and 0.30 ± 0.09 for the random groups.

**Fig. 3:**
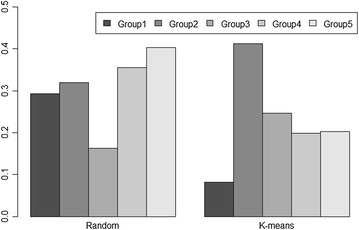
Prediction accuracies of direct genomic values for each random and K-means clustering cross-validation group according to the BayesA method

Accuracies within each cluster were also obtained using the method of Legarra et al. [38] and substantial differences between groups were observed with values ranging from 0.08 to 0.41 (Fig. 3). The lowest values were observed for K-means group 1, which was the most distinct cluster that included mainly Hereford animals (the zebu proportion was near zero) and showed the largest genetic distance to the other groups (Table 1). Therefore, this result is consistent with the fact that a reference population that includes only Braford cattle would not result in suitable accuracies for Hereford selection candidates [18]. Although all random groups had the same number of animals and the same genetic distance within and between clusters, accuracy for Group 3 was considerably lower (0.16) compared to the other random clusters. The highest accuracies were observed for K-means Group 5 (0.40) and random Group 2 (0.41).

Using the full set of 41,045 SNPs, pooled cross-validation accuracies for K-means and random clustering, respectively, were equal to 0.37 ± 0.08 and 0.43 ± 0.08 for BayesA, 0.37 ± 0.08 and 0.43 ± 0.08 for BayesB with π = 0.99, and 0.28 ± 0.07 and 0.40 ± 0.08 for BayesB with π = 0.999. When compared to the above results, accuracies that are derived using the proposed very low-density tag SNP panel with 58 SNPs would represent at least 68% of the accuracies of predictions obtained using all 41,045 SNPs with BayesB or BayesA methods. These results demonstrate that tag SNP panels may be used in commercial applications for genomic predictions in beef cattle as an alternative to more costly high-density panels. Nevertheless, the decision about the most suitable SNP density should be trait- and population-specific, depending on the relative accuracy and cost of the alternative SNP panels.

Cardoso et al. [18] reported pooled cross-validation accuracies of 0.39 and 0.44 for K-means and random clustering, respectively, for BayesB (π = 0.95) predictions obtained for tick count with the same population. These preview results obtained with a 50 K SNP panel represented accuracy gains of 50.0 and 51.7% when compared, respectively, to pedigree best linear unbiased prediction (PBLUP) accuracies of 0.26 for K-means and 0.29 for random groups obtained by the same authors. Compared to these results, the very low-density SNP panel that we propose here shows very similar accuracies to predictions based on conventional PBLUP. This would be the case for animals that are closely related to the reference population as in Cardoso et al. [18]. Even with similar accuracies, the very low-density panel predictions have the advantage of being applicable in the absence of historical tick count data, when phenotypes on ancestors may not be available, thus avoiding the need of population parasite burden. Moreover, blending strategies to combine tag SNP panel predictions with historical data from non-genotyped animals deserves further investigation, since they could improve prediction accuracies of selection candidates using, for example, single-step methodologies [62–64]. For these Hereford and Braford tick resistance datasets and the full set of SNPs, accuracy gains of using blended historical data by single-step genomic BLUP compared to Bayes B (π = 0.95) DGV were equal to 23 and 27% respectively for K-means and random cross-validation groups [18].

Bayesian approaches have been proposed for predicting genomic breeding values with high-density SNP panels, but in practice they may be more useful for low-density panels [65]. Decreased predictive abilities are expected for very low-density in comparison to high-density SNP panels, due to the expected reduced LD between SNPs and highly dispersed QTL affecting a particular trait. However, some studies have demonstrated the superiority of Bayesian methods to capture this LD between SNPs and QTL [66, 67]. Cleveland et al. [65] compared Bayesian prediction accuracies of different scenarios including high- and low-density SNP panels. These authors found similar accuracies when SNPs were selected based on the size of their additive effects, even when SNP coverage was extremely low, which corroborates our results. Dynamic schemes to successfully apply genomic selection technology for genetic improvement of livestock, invariably aim at minimizing genotyping costs while maximizing genetic gains and overall profits. Genotype data that are generated at lower costs from small subsets of highly informative SNPs could be used to genotype most of the animals in a herd and generate genomic breeding value predictions based on SNP effects that are estimated from high-density training datasets [68]. Moreover, our results show that recalculation of genetic effects for the most informative SNPs that were originally chosen from the full dataset resulted in the reduction of redundancy and/or confounding effects, which might have been included in estimates obtained during the original discovery using the 41,045 SNPs, as a result of multicollinearity among SNP effects. The applied strategy appears promising, since the obtained DGV retained about 70% of the accuracy of DGV derived from the full high-density panel, with only about 0.14% of the SNP density (58 out of 41,045). Similar strategies have already been proposed for predicting breeding values in young dairy cattle seedstock by using panels of about 3000 SNPs (larger than the panel proposed here) and resulted in accuracies representing 80 to 90% of those obtained with high-density panels with [69].

Genome-wide association studies allow for a much finer description of the genome and genomic selection results in increased genetic gains because early and accurate selection decisions are made possible, for traits that were previously ignored because of high associated phenotyping costs. Observed trends of decreasing genotyping costs in contrast to increasing expenses for phenotyping are expected to lead various livestock sectors to widely adopt genomic technology. However, the development of beef cattle training populations has been generally conducted by private companies and at a significantly slower pace compared to the dairy industry [17]. For a worldwide adoption of genomic selection in beef cattle breeding, it is still necessary to develop cost-effective strategies and robust training populations for more economically-relevant traits. Very low-density panels including informative SNPs may represent a viable alternative for including only one or a very few key complex traits of high economic value, such as tick resistance, that are not yet considered in traditional genetic evaluation, because they are too difficult or too costly to measure. These additional trait tag-SNP predictions could be combined with pedigree/phenotype-based breeding values that are regularly derived for production traits using selection index theory [70]. However, if genomic predictions require high prediction accuracies for many traits in a complex breeding goal, the tag-SNP panel strategy may not be effective due to a likely large number of SNPs in the panel when adding tag-SNPs across various traits.

Fine-mapping investigation using next-generation sequencing could also be used to target flanking regions around the currently identified tag SNPs, which may be involved in the biological mechanisms of tick resistance in Hereford and Braford cattle. The identification of causal mutations, along with the availability of a larger training population or suitable blending with historical data, would be decisive to propose cost-effective genomic evaluations based on very low-density marker panels to improve tick resistance in commercials herds.

## Conclusions

BayesB appears to be a suitable method for selecting tag SNPs based on Bayesian model frequency and *t*-like statistics. The resulting very low-density panel included SNPs that are potentially linked to functional gene networks and accounted for most of the genetic variance in tick resistance. The accuracy of genomic predictions derived from the proposed very low-density SNP panel using BayesA was moderate and may be useful for delivering cheaper genomic tests to the industry and for further studies related to fine-mapping for causal variants discovery.

## Additional file

**Additional file 1.** SNP name, reference sequence, chromosome and position (Chr_Pos), window coded number by GenSel in physical map order, description of the genes (symbol, database search, name, HGNC_id, genome which were mapped) mapped to the 58 SNPs selected from the GWAS analysis.
