# Supplementary material for: Tag SNP selection for prediction of tick resistance in Brazilian Braford and Hereford cattle breeds using Bayesian methods
Source: Genet Sel Evol. 2017 Jun 15;49:49. doi: 10.1186/s12711-017-0325-2 (PMC5471684; doi:10.1186/s12711-017-0325-2)
Supplement: Supplementary file 1 — Additional file 1. SNP name, reference sequence, chromosome and position (Chr_Pos), window coded number by GenSel in physical map order, description of the genes (symbol, database search, name, HGNC_id, genome which were mapped) mapped to the 58 SNPs selected from the GWAS analysis. [file 12711_2017_325_MOESM1_ESM.pdf]

Additional file 1: SNP Name, Reference Sequence, Chromosome and Position (Chr_Pos), window coded number by GenSel in physical map order, description of the genes (Symbol, database search, name, HGNC_id, genome which were mapped) mapped to the 58 SNP markers selected from the GWAS analysis.

| SNP_Name | SNP_RefSeq | Chr_Pos | Window | Gene_Symbol | Database | Gene_Name | HGNC_ID | Genome_map | Samp.[1] |
|---|---|---|---|---|---|---|---|---|---|
| BTB-01301015 | rs42429246 | 1_147255491 | 147 | PCBP3 | Ensembl | poly(rC) binding protein 3 | HGNC:8651 | Bos taurus | 5 |
| BTB-00077766 | rs43285940 | 2_4619367 | 163 | POLR2D | Ensembl | polymerase (RNA) II (DNA directed) polypeptide D | HGNC:9191 | Bos taurus | 5 |
| | | | | AMMECR1L | Ensembl | AMMECR1-like | HGNC:28658 | Bos taurus | |
| | | | | WDR33 | Ensembl | WD repeat domain 33 | HGNC:25651 | Bos taurus | |
| BTB-00082871 | rs43293394 | 2_20699862 | 179 | HOXD3 | Ensembl | homeobox D3 | HGNC:5137 | Bos taurus | 8 |
| | | | | MTX2 | Ensembl | metaxin 2 | HGNC:7506 | Bos taurus | |
| | | | | HOXD4 | Ensembl | homeobox D4 | HGNC:5138 | Bos taurus | |
| | | | | bta-mir-10b | Ensembl | miRBase | | Bos taurus | |
| | | | | HOXD1 | Ensembl | homeobox D1 | HGNC:5132 | Bos taurus | |
| ARS-BFGL-NGS-111213 | rs110504727 | 2_55308672 | 214 | LRP1B | NCBI | low density lipoprotein receptor-related protein 1B | HGNC:6693 | Bos taurus | 7 |
| BTB-01165311 | rs42320860 | 2_55283216 | 214 | LRP1B | NCBI | low density lipoprotein receptor-related protein 1B | HGNC:6693 | Bos taurus | 6 |
| Hapmap36094-SCAFFOLD96944_22403 | rs29026592 | 2_102214656 | 261 | SNORA11 | Ensembl | small nucleolar RNA, H/ACA box 11 | HGNC:32599 | Bos taurus | 4 |
| BTA-92857-no-rs | rs41662298 | 2_102723466 | 261 | SPAG16 | NCBI | sperm associated antigen 16 | HGNC:23225 | Bos taurus | 3 |
| ARS-BFGL-NGS-102874 | rs109276708 | 2_123152276 | 282 | SDC3 | Ensembl | syndecan 3 | HGNC:10660 | Bos taurus | 4 |
| | | | | PUM1 | Ensembl | pumilio RNA-binding family member 1 | HGNC:14957 | Bos taurus | |
| | | | | SNORD103 | Ensembl | Small nucleolar RNA SNORD103/SNORD85 | | Bos taurus | |
| BTB-00117780 | rs43328895 | 2_131393741 | 290 | CDC42 | Ensembl | cell division cycle 42 | HGNC:1736 | Bos taurus | 5 |
| | | | | U6 | Ensembl | RNA, U6 small nuclear 1 | HGNC:10227 | Bos taurus | |
| | | | | WNT4 | Ensembl | wingless-type MMTV integration site family, member 4 | HGNC:12783 | Bos taurus | |
| | | | | U6 | Ensembl | RNA, U6 small nuclear 50, pseudogene | HGNC:10229 | Bos taurus | |
| | | | | CELA3B | Ensembl | chymotrypsin-like elastase family, member 3B | HGNC:15945 | Bos taurus | |
| ARS-BFGL-NGS-113378 | rs43319306 | 2_134486603 | 293 | PAX7 | Ensembl | paired box 7 | HGNC:8621 | Bos taurus | 6 |
| | | | | TAS1R2 | Ensembl | taste receptor, type 1, member 2 | HGNC:14905 | Bos taurus | |

[1] Sampling number of each SNP within those ten cross-validation subsets proposed after GWAS and tag SNP selection strategy.

Additional file 1_cont: SNP Name, Reference Sequence, Chromosome and Position (Chr_Pos), window coded number by GenSel in physical map order, description of the genes (Symbol, database search, name, HGNC_id, genome which were mapped) mapped to the 58 SNP markers selected from the GWAS analysis.

| SNP_Name | SNP_RefSeq | Chr_Pos | Window | Gene_Symbol | Database | Gene_Name | HGNC_ID | Genome_map | Samp.[1] |
|---|---|---|---|---|---|---|---|---|---|
| ARS-BFGL-NGS-119309 | rs110043221 | 3_33691011 | 329 | EPS8L3 | Ensembl | EPS8-like 3 | HGNC:21297 | Bos taurus | 1 |
| | | | | GSTM1 | Ensembl | glutathione S-transferase mu 1 | HGNC:4632 | Bos taurus | |
| | | | | GSTM3 | Ensembl | glutathione S-transferase mu 3 (brain) | HGNC:4635 | Bos taurus | |
| | | | | CSF1 | Ensembl | colony stimulating factor 1 (macrophage) | HGNC:2432 | Bos taurus | |
| | | | | bta-mir-2413 | Ensembl | bta-mir-2413 (miRBase) | | Bos taurus | |
| ARS-BFGL-NGS-33433 | rs109299452 | 3_68207361 | 364 | LOC102724799 | NCBI | uncharacterized LOC102724799 | | Homo sapiens | 3 |
| BTB-00169573 | rs43385164 | 4_24209956 | 442 | | | | | | 4 |
| Hapmap45129-BTA-72713 | rs41587961 | 4_24932445 | 442 | ISPD | Ensembl | isoprenoid synthase domain containing | HGNC:37276 | Bos taurus | 4 |
| | | | | ISPD | Ensembl | isoprenoid synthase domain containing | HGNC:37276 | Bos taurus | |
| | | | | SOSTDC1 | Ensembl | sclerostin domain containing 1 | HGNC:21748 | Bos taurus | |
| ARS-BFGL-NGS-36591 | rs43403429 | 4_66830563 | 484 | FKBP14 | Ensembl | FK506 binding protein 14, 22 kDa | HGNC:18625 | Bos taurus | 4 |
| | | | | WIPF3 | Ensembl | WAS/WASL interacting protein family, member 3 | HGNC:22004 | Bos taurus | |
| | | | | SCRN1 | Ensembl | secernin 1 | HGNC:22192 | Bos taurus | |
| | | | | PLEKHA8 | Ensembl | pleckstrin homology domain containing, family A (phosphoinositide binding specific) member 8 | HGNC:30037 | Bos taurus | |
| Hapmap25270-BTA-142450 | rs109082916 | 4_97015274 | 515 | PLEX2 | Ensembl | PLEX2 (Bos taurus plexin A4 (PLXNA4), mRNA) | | Bos taurus | 4 |
| Hapmap22875-BTA-155031 | rs110223270 | 4_113721793 | 531 | ZNF775 | Ensembl | zinc finger protein 775 | HGNC:28501 | Bos taurus | 8 |
| | | | | GIMAP8 | Ensembl | GTPase, IMAP family member 8 | HGNC:21792 | Bos taurus | |
| | | | | GIMAP7 | Ensembl | GTPase, IMAP family member 7 | HGNC:22404 | Bos taurus | |
| Hapmap52967-rs29017027 | rs29017027 | 5_14044364 | 553 | DNAH5 | NCBI | dynein, axonemal, heavy chain 5 | | Homo sapiens | 8 |
| Hapmap30881-BTA-159706 | rs110661345 | 6_3994395 | 664 | QRFPR | Ensembl | pyroglutamylated RFamide peptide receptor | HGNC:15565 | Bos taurus | 4 |
| | | | | U6 | Ensembl | RNA, U6 small nuclear 1 | HGNC:10227 | Bos taurus | |
| | | | | U6 | Ensembl | RNA, U6 small nuclear 50, pseudogene | HGNC:10229 | Bos taurus | |
| BTB-01280976 | rs42404150 | 6_4193024 | 665 | QRFPR | Ensembl | pyroglutamylated RFamide peptide receptor | HGNC:15565 | Bos taurus | 5 |
| BTB-02002785 | rs43106284 | 6_49130874 | 710 | LOC100421158 | NCBI | piggyBac transposable element derived 4 pseudogene | | Homo sapiens | 9 |
| ARS-BFGL-NGS-111257 | rs110116877 | 7_13426119 | 794 | CACNA1A | Ensembl | calcium channel, voltage-dependent, P/Q type, alpha 1A subunit | HGNC:1388 | Bos taurus | 4 |

[1] Sampling number of each SNP within those ten cross-validation subsets proposed after GWAS and tag SNP selection strategy.

Additional file 1_cont: SNP Name, Reference Sequence, Chromosome and Position (Chr_Pos), window coded number by GenSel in physical map order, description of the genes (Symbol, database search, name, HGNC_id, genome which were mapped) mapped to the 58 SNP markers selected from the GWAS analysis.

| SNP_Name | SNP_RefSeq | Chr_Pos | Window | Gene_Symbol | Database | Gene_Name | HGNC_ID | Genome_map | Samp.[1] |
|---|---|---|---|---|---|---|---|---|---|
| ARS-BFGL-NGS-109534 | rs109115115 | 7_13608935 | 794 | TRMT1 | Ensembl | tRNA methyltransferase 1 homolog (S. cerevisiae) | HGNC:25980 | Bos taurus | 4 |
| | | | | NFIX | Ensembl | nuclear factor I/X (CCAAT-binding transcription factor) | HGNC:7788 | Bos taurus | |
| | | | | STX10 | Ensembl | syntaxin 10 | HGNC:11428 | Bos taurus | |
| | | | | NACC1 | Ensembl | nucleus accumbens associated 1 | HGNC:20967 | Bos taurus | |
| | | | | LYL1 | Ensembl | lymphoblastic leukemia associated hematopoiesis regulator 1 | HGNC:6734 | Bos taurus | |
| | | | | IER2 | Ensembl | immediate early response 2 | HGNC:28871 | Bos taurus | |
| BTB-01398754 | rs42522770 | 8_50525859 | 944 | LOC100422267 | NCBI | coagulation factor III (thromboplastin, tissue factor) pseudogene | | Homo sapiens | 7 |
| Hapmap40677-BTA-121871 | rs41622905 | 8_107821440 | 1001 | ASTN2 | NCBI | astrotactin 2 | HGNC:17021 | Bos taurus | 6 |
| ARS-BFGL-NGS-107048 | rs109728630 | 10_18395377 | 1132 | THSD4 | NCBI | thrombospondin, type I, domain containing 4 | HGNC:25835 | Bos taurus | 4 |
| Hapmap32096-BTA-150413 | rs110820714 | 10_46199529 | 1159 | HERC1 | Ensembl | HECT and RLD domain containing E3 ubiquitin protein ligase family member 1 | HGNC:4867 | Bos taurus | 7 |
| | | | | DAPK2 | Ensembl | death-associated protein kinase 2 | HGNC:2675 | Bos taurus | |
| ARS-BFGL-NGS-60054 | rs110382174 | 10_46571563 | 1159 | USP3 | Ensembl | ubiquitin specific peptidase 3 | HGNC:12626 | Bos taurus | 8 |
| Hapmap58695-rs29019899 | rs29019899 | 10_51735816 | 1164 | LIPC | Ensembl | lipase, hepatic | HGNC:6619 | Bos taurus | 10 |
| | | | | ADAM10 | Ensembl | ADAM metallopeptidase domain 10 | HGNC:188 | Bos taurus | |
| | | | | 5S_rRNA | Ensembl | 5S_rRNA (5S ribosomal RNA) | | Bos taurus | |
| ARS-BFGL-NGS-111871 | rs109034555 | 10_77105887 | 1190 | PLEKHG3 | Ensembl | pleckstrin homology domain containing, family G (with RhoGef domain) member 3 | HGNC:20364 | Bos taurus | 1 |
| | | | | SPTB | Ensembl | spectrin, beta, erythrocytic | HGNC:11274 | Bos taurus | |
| | | | | PPP1R36 | Ensembl | protein phosphatase 1, regulatory subunit 36 | HGNC:20097 | Bos taurus | |
| BTB-00464454 | rs43669951 | 11_21242337 | 1239 | MORN2 | Ensembl | MORN repeat containing 2 | HGNC:30166 | Bos taurus | 5 |
| | | | | SOS1 | Ensembl | son of sevenless homolog 1 (Drosophila) | HGNC:11187 | Bos taurus | |
| | | | | ARHGEF33 | Ensembl | Rho guanine nucleotide exchange factor (GEF) 33 | HGNC:37252 | Bos taurus | |
| | | | | DHX57 | Ensembl | DEAH (Asp-Glu-Ala-Asp/His) box polypeptide 57 | HGNC:20086 | Bos taurus | |
| | | | | GEMIN6 | Ensembl | gem (nuclear organelle) associated protein 6 | HGNC:20044 | Bos taurus | |

[1] Sampling number of each SNP within those ten cross-validation subsets proposed after GWAS and tag SNP selection strategy.

Additional file 1_cont: SNP Name, Reference Sequence, Chromosome and Position (Chr_Pos), window coded number by GenSel in physical map order, description of the genes (Symbol, database search, name, HGNC_id, genome which were mapped) mapped to the 58 SNP markers selected from the GWAS analysis.

| SNP_Name | SNP_RefSeq | Chr_Pos | Window | Gene_Symbol | Database | Gene_Name | HGNC_ID | Genome_map | Samp.[1] |
|---|---|---|---|---|---|---|---|---|---|
| Hapmap60779-rs29022104 | rs29022104 | 11_65975590 | 1283 | LOC100420020 | NCBI | transmembrane protein 55B pseudogene | | Homo sapiens | 7 |
| ARS-BFGL-NGS-111179 | rs110144789 | 11_101217080 | 1319 | FIBCD1 | Ensembl | fibrinogen C domain containing 1 | HGNC:25922 | Bos taurus | 9 |
| | | | | QRFP | Ensembl | pyroglutamylated RFamide peptide | HGNC:29982 | Bos taurus | |
| | | | | LAMC3 | Ensembl | laminin, gamma 3 | HGNC:6494 | Bos taurus | |
| | | | | ABL1 | Ensembl | ABL proto-oncogene 1, non-receptor tyrosine kinase | HGNC:76 | Bos taurus | |
| Hapmap44228-BTA-34185 | rs111003257 | 13_14814734 | 1429 | | | | | | 7 |
| Hapmap40517-BTA-33731 | rs41577070 | 13_73782308 | 1488 | snoU2_19 | Ensembl | Small nucleolar RNA U2-19 | | Bos taurus | 6 |
| | | | | U2 | Ensembl | RNA, U2 small nuclear 5, pseudogene | HGNC:10155 | Bos taurus | |
| | | | | U2 | Ensembl | RNA, U2 small nuclear 1 | HGNC:10142 | Bos taurus | |
| | | | | ADA | Ensembl | adenosine deaminase | HGNC:186 | Bos taurus | |
| | | | | WISP2 | Ensembl | WNT1 inducible signaling pathway protein 2 | HGNC:12770 | Bos taurus | |
| | | | | U2 | Ensembl | RNA, U2 small nuclear 2, pseudogene | HGNC:10152 | Bos taurus | |
| | | | | PKIG | Ensembl | protein kinase (cAMP-dependent, catalytic) inhibitor gamma | HGNC:9019 | Bos taurus | |
| | | | | U2 | Ensembl | RNA, U2 small nuclear 4, pseudogene | HGNC:10154 | Bos taurus | |
| | | | | RIMS4 | Ensembl | regulating synaptic membrane exocytosis 4 | HGNC:16183 | Bos taurus | |
| | | | | KCNK15 | Ensembl | potassium channel, two pore domain subfamily K, member 15 | HGNC:13814 | Bos taurus | |
| | | | | U2 | Ensembl | RNA, U2 small nuclear 3, pseudogene | HGNC:10153 | Bos taurus | |
| | | | | snoU2-30 | Ensembl | Small nucleolar RNA U2-30 | | Bos taurus | |
| BTA-33938-no-rs | rs41567254 | 13_80350185 | 1495 | SALL4 | Ensembl | spalt-like transcription factor 4 | HGNC:15924 | Bos taurus | 6 |
| | | | | ATP9A | Ensembl | ATPase, class II, type 9A | HGNC:13540 | Bos taurus | |
| ARS-BFGL-NGS-112197 | rs110051873 | 13_80563915 | 1495 | ZFP64 | Ensembl | ZFP64 zinc finger protein | HGNC:15940 | Bos taurus | 6 |
| Hapmap41120-BTA-99310 | rs41668729 | 13_80980304 | 1495 | LOC102724139 | NCBI | uncharacterized LOC102724139 | | Homo sapiens | 7 |
| Hapmap57013-rs29019369 | rs29019369 | 13_70790140 | 1485 | CHD6 | Ensembl | chromodomain helicase DNA binding protein 6 | HGNC:19057 | Bos taurus | 6 |
| | | | | bta-mir-544b-1 | Ensembl | miRBase | | Bos taurus | |

[1] Sampling number of each SNP within those ten cross-validation subsets proposed after GWAS and tag SNP selection strategy.

Additional file 1_cont: SNP Name, Reference Sequence, Chromosome and Position (Chr_Pos), window coded number by GenSel in physical map order, description of the genes (Symbol, database search, name, HGNC_id, genome which were mapped) mapped to the 58 SNP markers selected from the GWAS analysis.

| SNP_Name | SNP_RefSeq | Chr_Pos | Window | Gene_Symbol | Database | Gene_Name | HGNC_ID | Genome_map | Samp.[1] |
|---|---|---|---|---|---|---|---|---|---|
| BTB-00915241 | rs42075995 | 14_54323858 | 1553 | CSMD3 | Ensembl | CUB and Sushi multiple domains 3 | HGNC:19291 | Bos taurus | 10 |
| ARS-BFGL-NGS-5811 | rs110197574 | 15_37534610 | 1621 | RPS15P8 | NCBI | ribosomal protein S15 pseudogene 8 | | Homo sapiens | 11 |
| Hapmap51782-BTA-95909 | rs41665212 | 15_37575605 | 1621 | RPS15P8 | NCBI | ribosomal protein S15 pseudogene 8 | | Homo sapiens | 9 |
| Hapmap45825-BTA-25376 | rs41629346 | 15_72980544 | 1656 | NEO1 | NCBI | neogenin 1 | | Homo sapiens | 4 |
| ARS-BFGL-NGS-40365 | rs41257804 | 16_40059619 | 1709 | MYOC | Ensembl | myocilin, trabecular meshwork inducible glucocorticoid response | HGNC:7610 | Bos taurus | 8 |
| | | | | VAMP4 | Ensembl | vesicle-associated membrane protein 4 | HGNC:12645 | Bos taurus | |
| | | | | DNM3 | Ensembl | dynamin 3 | HGNC:29125 | Bos taurus | |
| | | | | METTL13 | Ensembl | methyltransferase like 13 | HGNC:24248 | Bos taurus | |
| BTB-01197909 | rs42354418 | 16_40086288 | 1709 | VAMP4 | Ensembl | vesicle-associated membrane protein 4 | HGNC:12645 | Bos taurus | 6 |
| | | | | METTL13 | Ensembl | methyltransferase like 13 | HGNC:24248 | Bos taurus | |
| | | | | DNM3 | Ensembl | dynamin 3 | HGNC:29125 | Bos taurus | |
| Hapmap48746-BTA-40116 | rs41583441 | 16_74283537 | 1743 | KCNH1 | Ensembl | potassium channel, voltage gated eag related subfamily H, member 1 | HGNC:6250 | Bos taurus | 3 |
| ARS-BFGL-BAC-27352 | rs109822497 | 17_7756498 | 1758 | DCLK2 | Ensembl | doublecortin-like kinase 2 | HGNC:19002 | Bos taurus | 4 |
| Hapmap34041-BES1_Contig298_838 | rs43708490 | 20_17837675 | 1974 | ZSWIM6 | Ensembl | zinc finger, SWIM-type containing 6 | HGNC:29316 | Bos taurus | 9 |
| Hapmap28040-BTA-134983 | rs109850399 | 20_19917959 | 1976 | 5S_rRNA | Ensembl | 5S_rRNA (5S ribosomal RNA) | | Bos taurus | 5 |
| Hapmap43377-BTA-85612 | rs41660483 | 20_56196291 | 2013 | U6 | Ensembl | RNA, U6 small nuclear 50, pseudogene | HGNC:10229 | Bos taurus | 4 |
| | | | | MYO10 | Ensembl | myosin X | HGNC:7593 | Bos taurus | |
| | | | | SNORA9 | Ensembl | small nucleolar RNA, H/ACA box 9 | HGNC:32597 | Bos taurus | |
| | | | | U6 | Ensembl | RNA, U6 small nuclear 1 | HGNC:10227 | Bos taurus | |
| ARS-BFGL-NGS-13702 | rs109546362 | 20_71498820 | 2028 | TPPP | Ensembl | tubulin polymerization promoting protein | HGNC:24164 | Bos taurus | 8 |
| | | | | NKD2 | Ensembl | naked cuticle homolog 2 (Drosophila) | HGNC:17046 | Bos taurus | |
| | | | | BRD9 | Ensembl | bromodomain containing 9 | HGNC:25818 | Bos taurus | |
| | | | | SLC12A7 | Ensembl | solute carrier family 12 (potassium/chloride transporter), member 7 | HGNC:10915 | Bos taurus | |
| | | | | TRIP13 | Ensembl | thyroid hormone receptor interactor 13 | HGNC:12307 | Bos taurus | |

[1] Sampling number of each SNP within those ten cross-validation subsets proposed after GWAS and tag SNP selection strategy.

Additional file 1_cont: SNP Name, Reference Sequence, Chromosome and Position (Chr_Pos), window coded number by GenSel in physical map order, description of the genes (Symbol, database search, name, HGNC_id, genome which were mapped) mapped to the 58 SNP markers selected from the GWAS analysis.

| SNP_Name | SNP_RefSeq | Chr_Pos | Window | Gene_Symbol | Database | Gene_Name | HGNC_ID | Genome_map | Samp.[1] |
|---|---|---|---|---|---|---|---|---|---|
| BTB-00849206 | rs42019145 | 22_45866182 | 2146 | ERC2 | NCBI | ELKS/RAB6-interacting/CAST family member 2 | HGNC:31922 | Bos taurus | 5 |
| ARS-BFGL-NGS-84660 | rs109886577 | 25_15365566 | 2294 | | | | | | 4 |
| ARS-BFGL-BAC-37178 | rs42066295 | 25_30062438 | 2309 | AUTS2 | Ensembl | autism susceptibility candidate 2 | HGNC:14262 | Bos taurus | 3 |
| Hapmap48932-BTA-88109 | rs41663418 | 28_20480901 | 2440 | | | | | | 6 |
| BTB-01129090 | rs43733032 | 28_20915841 | 2440 | | | | | | 6 |
| Hapmap34915-BES7_Contig278_1082 | rs43702480 | 28_20988266 | 2440 | | | | | | 5 |
| BTA-66199-no-rs | rs43707003 | 29_48735363 | 2515 | SHANK2 | NCBI | SH3 and multiple ankyrin repeat domains 2 | HGNC:14295 | Bos taurus | 4 |

[1] Sampling number of each SNP within those ten cross-validation subsets proposed after GWAS and tag SNP selection strategy.
